# Effect of inulin, galacto oligosaccharides and iron fortification on iron deficiency anemia among women of reproductive age; a randomized controlled trial

**DOI:** 10.3389/fnut.2022.1028956

**Published:** 2022-11-14

**Authors:** Sehar Iqbal, Waqas Ahmed, Saira Zafar, Umar Farooq, Juweria Abid, Hassan Bin Usman Shah, Sajeela Akram, Madiha Ghazanfar, Abdul Momin Rizwan Ahmad

**Affiliations:** ^1^College of Pharmacy, Al-Ain University, Abu Dhabi, United Arab Emirates; ^2^Department of Food Science and Human Nutrition, University of Veterinary and Animal Sciences, Lahore, Pakistan; ^3^Department of Public Health, Health Services Academy, Islamabad, Pakistan; ^4^Department of Diet and Nutritional Sciences, IBADAT International University, Islamabad, Pakistan; ^5^Department of Nutrition and Dietetics, National University of Medical Sciences, Rawalpindi, Pakistan; ^6^The Kirby Institute, University of New South Wales, Sydney, NSW, Australia; ^7^Department of Human Nutrition and Dietetics, University of Chakwal, Chakwal, Pakistan

**Keywords:** iron deficiency anemia, prebiotics, inulin, galacto oligosaccharides, iron fortification

## Abstract

Iron supplementation and fortification are the well-known approaches to treat iron deficiency anemia (IDA) in women of reproductive age. The objective of the current randomized controlled trial (RCT) was to evaluate the cumulative effects of prebiotics and iron fortification among women of reproductive age. For this purpose, a total of 75 iron deficient women of childbearing age were recruited and randomly divided into 5 groups (4 treatment groups and 1 control group). Four different types of fortified wheat flour were prepared using two iron fortificants (NaFeEDTA and FeSO_4_) and two prebiotics [inulin and galacto oligosaccharides (GOS)], while control group was treated with iron fortified flour without any prebiotics. Blood samples were collected from overnight fasted women on monthly basis up to 90 days. Hematological indices such as Hemoglobin (Hb), Hematocrit, Red Blood Cell (RBC) Count and Mean Corpuscular Volume (MCV), as well as iron biomarkers including serum iron, ferritin, transferrin, and Total Iron Binding Capacity (TIBC) were evaluated for analyses. The results showed a considerable positive improvement in all iron biomarkers as well as hematological indices among the treatment groups (*P*-value < 0.05), as compared to the control group. A maximum Hb (11.86 ± 0.24 mg/dL) and hematocrit value (35.06 ± 1.32%), was reported in group G_3_ which was treated with fortified wheat flour at a dose of 963 mg/kg GOS + 15 ppm FeSO_4_. On the other hand, highest mean values for RBC Count (4.73 ± 0.41 mil/mm^3^), MCV (81.41 ± 3.21 fL), serum iron (75.62 ± 2.79 μg/dL), serum transferrin (16.82 ± 0.30 mg/dL), and TIBC (403.68 ± 7.27 μg/dL) were observed in G_4_ group receiving the fortified wheat flour at a dose of 963 mg/kg GOS + 30 ppm FeSO_4_ level. The study concluded that prebiotic fortification along with iron salts helps to enhance iron absorption among iron deficiency anemic women of reproductive age.

## Introduction

Iron, being an important constituent of certain proteins and several enzymes plays a major role in energy metabolism, oxygen transport, ATP synthesis, and biochemical transformation (1). However, iron deficiency is one amongst the major public health issues responsible to increase the global burden of disease, mainly affecting children and women (2). Such as, iron deficiency is a leading cause of infections, cognitive impairment, reduced work capacity, and high mortality risks in women of reproductive age (3). Besides, iron deficiency anemia during pregnancy has been reported to increase the risk of pregnancy complications and poor perinatal outcomes in low-and-middle income countries (4). Before conception, a woman should have adequate iron stores primarily required for the feto-placental development (∼360 mg Fe), expansion of red blood cells (450 mg Fe), and blood lost at delivery (∼150 mg), but unfortunately many women have insufficient preconception iron stores to meet these pregnancy needs (5). Recent estimates show that approximately 36.5% pregnant women and over half a billion women of reproductive age (15–49 years) are suffering from anemia (6). Considering these alarming figures, the World Health Organization (WHO) emphasizes on the target of 50% reduction in anemia by 2025 through improvement of dietary diversity, food fortification and iron supplementation (7).

Eating food processed from animal liver and animal sources during pregnancy is the safest and most effective method of iron supplementation. Food fortification with iron is the other well-known approach to treat anemia in women of reproductive age (8). Addition of vitamins and minerals through fortification helps to improve food quality and nutritional value. Equally, studies have shown that prebiotics such as galacto oligosaccharides and inulin might play an important role in enhancing iron absorption in anemic subjects (9). A recent study reported that co-administration of inulin and iron fortificants helps to improve iron biomarkers in female anemic rats (10). In concomitant, few studies suggested that Oligofructose helps to prevent iron deficiency anemia by increasing iron absorption in the gut (11, 12).

Prebiotics are known as functional food components that stimulate the growth and colonization of beneficial bacteria in the gut. These microorganisms scavenge iron from the environment though producing high/specific affinity iron-chelating compounds (siderophores system) (13). Furthermore, prebiotics stimulate the absorption of iron in proximal colon as well as duodenum through producing short chain fatty acids in the colon (14). Thus absorption of iron is enhanced in the presence of fermentable food involved in the growth of bacteria leading to the production of short-chain fatty acids such as propionic acid (13). However, scarcity of data and research on human subject warrants further studies in this regard considering high prevalence of IDA in low/middle income countries.

Pakistan being a low/middle income country is facing the major public health issue of iron deficiency in children and women of reproductive age. According to the latest estimates, approximately 42% women of reproductive age and more than half (56.6%) of adolescent girls are suffering with anemia in Pakistan (15). The current study therefore aimed to provide research-based evidence to target the preventive approach and minimizing the risk of IDA in the country. Thus, the objective of this randomized control trial (RCT) was to assess the cumulative effect of prebiotics and iron fortification on hematological indices and serum iron biomarkers among women of reproductive age group. Hemoglobin (Hb), Hematocrit, Red Blood Cell (RBC) Count and Mean Corpuscular Volume (MCV), as well as iron biomarkers including serum iron, ferritin, transferrin and Total Iron Binding Capacity (TIBC) were evaluated to observe the combined effects of prebiotics and iron fortification on iron deficiency anemic women.

## Materials and methods

### Study design

It was a double blind, randomized controlled trial which lasted for 3 months. For this purpose, the study population of university going iron deficient female adults (age 18–25 years) were screened based on the signs and symptoms associated with iron deficiency anemia. Later, these short-listed participants went for serum ferritin and complete blood count (CBC) tests to confirm diagnosis and to have baseline values. The participants were recruited from the three federal universities of Islamabad, Pakistan. An informed written consent was taken from all the study subjects prior to the start of RCT.

### Study participants

**FIGURE 1:**
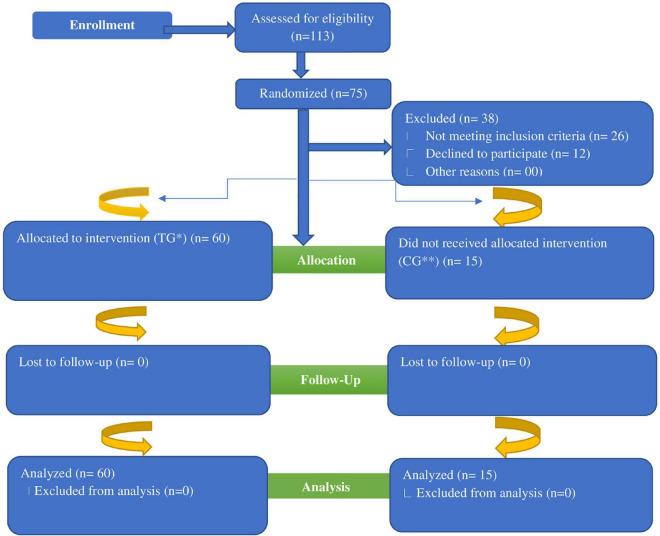
Flow diagram of women subjects through the randomized controlled trial. *TG, Treatment Group; **CG, Control Group.

A total of 113 women subjects were initially assessed for detection of iron deficiency (Figure 1). Out of these 113 shortlisted participants, 38 were excluded (26 did not meet the eligibility criteria whereas 12 participants refused to participate in the study). Remaining (75 participants) were finally recruited for this RCT. The flow chart of randomization has been presented in Figure 1. The selected participants underwent for a complete comprehensive medical examination to exclude the possibility of any confounders such as suffering from any chronic and GI disorders. Finally, 75 selected study subjects were divided into 5 groups comprising of 4 treatment groups and 1 control group (Table 1). The control group received only iron fortification without addition of any prebiotics. The current RCT continued from March 2019 to June 2019, while eligibility of the included participants started in January 2019 based on the convenience sampling technique.

**TABLE 1:** Treatment plan (iron fortificants and prebiotics based diet).

Groups	Diet plan	Human equivalent dose (HED) for prebiotic
G_0_	Control (no prebiotic given)	–
G_1_	963 mg/kg Inulin + 10 ppm NaFeEDTA	133 mg/kg = 8 g
G_2_	963 mg/kg Inulin + 20 ppm NaFeEDTA	133 mg/kg = 8 g
G_3_	963 mg/kg GOS + 15 ppm FeSO_4_	133 mg/kg = 8 g
G_4_	963 mg/kg GOS + 30 ppm FeSO_4_	133 mg/kg = 8 g

### Inclusion and exclusion criteria

All willing iron deficient female adults without any chronic diseases such as diabetes or hypertension were included in the study. On the other hand, women suffering from any chronic diseases, taking iron and/or prebiotic supplements, smokers and married or having children were excluded from the study.

### Randomization and blinding

Simple randomization of study participants into groups was done *via* using computer generated random number tables. Since the study was double blinded, neither study participants nor data collectors in contact with the selected study subjects were aware about the type of fortified flour, allocation sequence of wheat flours, or method of preparing prebiotics and iron fortified flours used for this trial.

### Diet plan

Prebiotics and iron fortificants required for this study were bought from the well reputed company of “Sigma Aldrich.” Proper storage was ensured as per the instructions on the label for each ingredient. In this study, two different types of iron fortificants “NaFeEDTA and FeSO_4_^”^ were added at two different levels at 10 ppm and 20 ppm NaFeEDTA and 15 ppm and 30 ppm FeSO_4_. Similarly, two different prebiotics “Inulin and Galacto oligosaccharides” both at a level of 963 mg/kg body weight were used for current study.

The dosage for prebiotics was calculated using the human equivalent dose (HED) equation, that is,


HED⁢(mg/kg)=Animal⁢Dose⁢in⁢mg/kg×(AnimalWeightinkgs)0.33(Human⁢Weight⁢in⁢kgs)

Recommended dose of prebiotics for human consumption is 6–8 grams per day. Sample calculations for reference weight of 60 kgs human, 0.15 kg rat and 8 grams prebiotic consumption per day were performed as follows;

HED = 963 × (0.15/60)^0.33^
HED = 963 × (0.0025)^0.33^
HED = 963 × 0.1384
HED = 133.33 mg/kg/day
HED = 133.33 × 60
HED = 7999.8 ≈ 8 g per day

Exact dosage given to the study subjects was however calculated after weighing each study subject individually. Moreover, dosage was adjusted at the end of each week, according to the changes in body weight of each study participant.

### Experimental protocol

For fortification, wheat flour was selected considering the major staple food consumed in Pakistan on daily basis by the wider regional/local community. Four different types of wheat flour were prepared based on the calculated dose of prebiotic as well as added iron fortificant (Table 1).

Wheat flours were prepared on weekly basis and participants were given the prepared flour for the subsequent week on each Sunday night. Participants were advised not to drink tea or coffee within 30 min after consumption of the fortified wheat flour.

### Efficacy trials

For a period of 3 months, blood samples were collected from overnight fasted women on monthly basis. Four readings were taken overall, that is, at baseline, 30th day, 60th day, and 90th day.

### Analytical procedures

Blood samples were collected from overnight fasted women and subjected to various analytical procedures.

#### Hematological indices

Four types of hematological indices Hb, Hematocrit, RBC Count and MCV were analyzed through automated cell counter. For determination of hemoglobin and MCV, automated hematology analyzer was used while hematocrit was determined as the ratio of the volume of packed red blood cells to the total blood volume. For RBC Count, flow cytometry technique was used (16).

#### Iron biomarkers

In addition to the hematological indices, four different iron biomarkers including serum iron, ferritin, transferrin, and TIBC were also evaluated on monthly basis up to 90 days of study duration.

##### Serum iron

Serum iron concentration was determined by Atomic Absorption Spectrophotometer. To determine serum iron, blood was centrifuged at 5,000 rpm followed by freezing of serum at –20°C. Serum iron was then measured using Atomic Absorption Spectrometry (17).

##### Serum ferritin

Commercially available kit of Ciba-Corning’s Automated Chemiluminescence System’s (ACS 180) was used to evaluate the levels of serum ferritin among anemic subjects. Serum ferritin levels were determined using the technique of Electrochemiluminescence immunoassay (ECLIA) (18).

##### Serum transferrin

Serum Transferrin was determined in anemic women as per the protocol described by Al-Buhairan and Oluboyede. For analysis of serum transferrin, Cobas Mira Plus Analyzer was used. The specific antiserum formed by Human transferrin, was measured at 340 nm turbidimetrically (19).

##### Total iron binding capacity

Total Iron Binding Capacity was calculated using the standard formula, that is, TIBC = Transferrin × 24.

### Statistical analysis

Factorial design was employed to determine the level of significance using SPSS version 23.0. Data was considered significant at *P*-value < 0.05 (20). All the results were presented as Means and Standard Deviations.

## Results

### Effect of prebiotic and iron fortified diet on hematological indices and iron biomarkers

Mean square values for hemoglobin, hematocrit, RBC Count, MCV, serum iron, serum ferritin, Serum transferrin, and TIBC showed significant positive variations for the effect of groups, study intervals as well as their interaction (Table 2).

**TABLE 2:** Mean squares regarding hematological indices and iron biomarkers for anemic women fed with prebiotic and iron fortified diet.

SOV	df	Hematological indices				Iron biomarkers			
		Hemoglobin (mg/dL)	Hematocrit (%)	RBC count (mil/mm^3^)	MCV (fL)	Serum iron (μg/dL)	Serum ferritin (ng/mL)	Serum transferrin (mg/dL)	Total iron binding capacity (μg/dL)
Groups	4	0.49*	83.03*	2.04*	968.19*	306.96*	77.44*	2.71*	1556.81*
Study intervals	3	4.46*	203.41*	4.54*	1036.61*	266.32*	96.03*	2.10*	1232.31*
Groups × study intervals	12	0.22*	5.82*	0.17*	10.10*	5.38*	8.60*	0.08*	46.21*
Error	280	0.09	0.06	0.09	0.03	0.38	1.92	0.05	3.23
Total	299								

*Significant (*P*-value < 0.05).

### Hemoglobin

Mean values for hemoglobin levels along with their respective standard deviations have been shown in Table 3.

**TABLE 3:** Effect of fortified diets on hemoglobin levels (mg/dL) among anemic women.

Treatments/Groups	Days				Means
	0	30	60	90	
G_0_	11.44 ± 0.04^c^	11.52 ± 0.02^d^	11.64 ± 0.03^d^	11.91 ± 0.02^d^	11.62 ± 0.21^c^
G_1_	11.58 ± 0.02^a^	11.71 ± 0.01^b^	11.83 ± 0.04^b^	12.04 ± 0.03^c^	11.79 ± 0.20^ab^
G_2_	11.54 ± 0.01^b^	11.62 ± 0.03^c^	11.74 ± 0.02^c^	11.85 ± 0.04^e^	11.68 ± 0.14^bc^
G_3_	11.61 ± 0.02^a^	11.78 ± 0.03^a^	11.90 ± 0.01^a^	12.17 ± 0.04^b^	11.86 ± 0.24^a^
G_4_	11.28 ± 0.04^d^	11.54 ± 0.02^d^	11.84 ± 0.01^b^	12.33 ± 0.03^a^	11.74 ± 0.45^b^

Means carrying the same letters in a column are not significantly different. G_0_ = Control Group (no prebiotic given), G_1_ = 963 mg/kg Inulin + 10 ppm NaFeEDTA, G_2_ = 963 mg/kg Inulin + 20 ppm NaFeEDTA, G_3_ = 963 mg/kg GOS + 15 ppm FeSO_4_, G_4_ = 963 mg/kg GOS + 30 ppm FeSO_4_.

The results showed the maximum Hb value of 11.86 ± 0.24 mg/dL in group G_3_ which was given the fortified wheat dose at 963 mg/kg GOS + 15 ppm FeSO_4_, followed by the Hb value of G_1_ group at 11.79 ± 0.20 mg/dL, G_4_ at 11.74 ± 0.45 mg/dL and G_2_ at 11.68 ± 0.14 mg/dL. The control group G_0_ reported with the mean value for hemoglobin at 11.62 ± 0.21 mg/dL.

As the study intervals progressed, a significant improvement was seen in the mean hemoglobin levels of anemic women over the period of 90 days. Maximum Hb improvement could be seen in group G_4_ ranged from 11.28 ± 0.04 mg/dL at the start of the study to 12.33 ± 0.03 mg/dL at the end of the study. This was followed by group G_3_ in which the variation of the levels reported from 11.61 ± 0.02 mg/dL to 12.17 ± 0.04 mg/dL. The subsequent improvement was observed in groups G_0_, G_1_ and G_2_ where the hemoglobin values varied from 11.44 ± 0.04 mg/dL, 11.58 ± 0.02 mg/dL and 11.54 ± 0.01 mg/dL at 0 day to 11.91 ± 0.02 mg/dL, 12.04 ± 0.03 mg/dL and 11.85 ± 0.04 mg/dL at 90th day, respectively.

### Hematocrit

According to the mean values of Hematocrit, the maximum value of 35.06 ± 1.32% was seen in group G_3_, followed by group G_1_ (35.04 ± 1.10%), group G_4_ (34.60 ± 2.66%), respectively (Table 4). On the other hand, the control group G_0_ showed a mean value of 32.22 ± 1.73%.

**TABLE 4:** Effect of fortified diets on hematocrit levels (%) among anemic women.

Treatments/Groups	Days				Means
	0	30	60	90	
G_0_	30.26 ± 0.17^e^	31.62 ± 0.04^d^	32.65 ± 0.03^d^	34.36 ± 0.16^d^	32.22 ± 1.73^b^
G_1_	33.76 ± 0.19^a^	34.84 ± 0.12^a^	35.14 ± 0.10^b^	36.43 ± 0.07^b^	35.04 ± 1.10^a^
G_2_	32.77 ± 0.07^c^	33.72 ± 0.08^c^	34.76 ± 0.17^c^	36.17 ± 0.07^c^	34.35 ± 1.46^a^
G_3_	33.50 ± 0.06^b^	34.59 ± 0.09^b^	35.60 ± 0.07^a^	36.56 ± 0.06^b^	35.06 ± 1.32^a^
G_4_	31.50 ± 0.06^d^	33.81 ± 0.13^c^	35.24 ± 0.06^b^	37.86 ± 0.26^a^	34.60 ± 2.66^a^

Means carrying the same letters in a column are not significantly different. G_0_ = Control Group (no prebiotic given), G_1_ = 963 mg/kg Inulin + 10 ppm NaFeEDTA, G_2_ = 963 mg/kg Inulin + 20 ppm NaFeEDTA, G_3_ = 963 mg/kg GOS + 15 ppm FeSO_4_, G_4_ = 963 mg/kg GOS + 30 ppm FeSO_4_.

With the progression of study duration, maximum improvement was observed in group G_4_, followed by group G_2_, G_3_ and G_1_. For group G_4_, the hematocrit levels improved from 31.50 ± 0.06 at 0 day to 37.86 ± 0.26% at 90th day while for group G_2_, the improvement in hematocrit levels was 32.77 ± 0.07–36.17 ± 0.07%. In group G_3_, the hematocrit level enhanced from 33.50 ± 0.06 to 36.56 ± 0.06%, while Group G_1_ had a value of 33.76 ± 0.19% at the start and improved to 36.43 ± 0.07% at the end of 90 days.

### Red blood cell count

**FIGURE 2:**
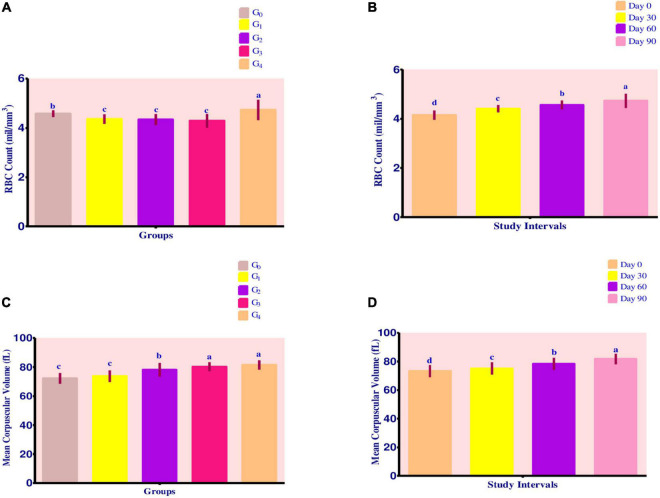
**(A–D)** Effect of fortified diets on RBC count and MCV among anemic women data are presented as Means ± *SD*. *P*-value < 0.05 is considered to be significant. Means carrying the same letters are not significantly different.

Mean values for RBC Count (shown in Figures 2A,B) demonstrated a maximum value in group G_4_, followed by groups G_1_, G_2_, and G_3_. The recorded values for groups G_4_, G_1_, G_2_, and G_3_ were 4.73 ± 0.41 mil/mm^3^, 4.36 ± 0.19 mil/mm^3^, 4.34 ± 0.22 mil/mm^3^ and 4.29 ± 0.28 mil/mm^3^, respectively.

Over the course of 90 days, RBC count levels steadily increased. Maximum improvement was recorded in groups G_4_, G_3_, G_2_, and G_1_, respectively. For group G_4_, change in RBC count levels was from 4.26 ± 0.02 mil/mm^3^ to 5.23 ± 0.02 mil/mm^3^. For group G_3_, the levels improved from 3.92 ± 0.03 mil/mm^3^ to 4.56 ± 0.02 mil mm^3^, whereas RBC count levels increased from 4.04 ± 0.02 mil/mm^3^ to 4.57 ± 0.01 mil/mm^3^ for group G_2_. Group G_1_ had a value of 4.13 ± 0.02 mil/mm^3^ improved to 4.57 ± 0.01 mil/mm^3^ at the end of 90-day trial period.

### Mean corpuscular volume

Mean values for MCV showed a steady improvement (Figure 2C). Maximum value for MCV was attained by group G_4_ (81.41 ± 3.21 fL), followed by G_3_ 80.21 ± 3.13 fL, G_2_ 78.11 ± 4.71 fL, and G_1_ 73.73 ± 3.99 fL. On the other hand, control group G_0_ showed a MCV value of 72.20 ± 3.68 fL.

Across the modeling trials, MCV levels improved steadily as shown by Figure 2D. With the progress in study intervals, maximum improvement occurred in group G_2_, followed by group G_1_, G_0_, G_4_, and G_3_. For group G_2_, the MCV levels ranged from 73.79 ± 0.19 fL at 0 day to 83.77 ± 0.13 fL at 90th day.

### Serum iron

Table 5 shows that group G_4_ had a value of 75.62 ± 2.79 μg/dL while group G_2_ had a value of 74.70 ± 2.06 μg/dL. Group G_3_ recorded a value of 73.38 ± 2.04 μg/dL while group G_1_ was observed having a value of 73.07 ± 1.97 μg/dL. Group G_0_ showed a value of 69.69 ± 1.12 μg/dL.

**TABLE 5:** Effect of fortified diets on serum iron levels (μg/dL) among anemic women.

Treatments/Groups	Days				Means
	0	30	60	90	
G_0_	68.41 ± 0.70^c^	69.17 ± 0.85^d^	70.25 ± 0.85^d^	70.93 ± 0.95^e^	69.69 ± 1.12^d^
G_1_	70.97 ± 0.70^b^	72.34 ± 1.01^c^	74.34 ± 1.12^c^	75.36 ± 1.23^d^	73.07 ± 1.97^c^
G_2_	72.65 ± 0.50^a^	73.95 ± 0.77^b^	75.50 ± 0.90^b^	77.42 ± 0.91^b^	74.70 ± 2.06^b^
G_3_	71.41 ± 0.60^b^	72.62 ± 0.73^c^	73.96 ± 0.99^c^	76.16 ± 1.09^c^	73.38 ± 2.04^c^
G_4_	72.60 ± 0.33^a^	74.51 ± 0.39^a^	76.61 ± 0.53^a^	79.10 ± 0.95^a^	75.62 ± 2.79^a^

Means carrying the same letters in a column are not significantly different. G_0_ = Control Group (no prebiotic given), G_1_ = 963 mg/kg Inulin + 10 ppm NaFeEDTA, G_2_ = 963 mg/kg Inulin + 20 ppm NaFeEDTA, G_3_ = 963 mg/kg GOS + 15 ppm FeSO_4_, G_4_ = 963 mg/kg GOS + 30 ppm FeSO_4_.

Across the modeling trials, a significant improvement was witnessed from the time of initiation to the time of termination at 90th day. Maximum improvement in the trait was witnessed in group G_4_, with value ranging from 72.60 ± 0.33 μg/dL to 79.10 ± 0.95 μg/dL. In group G_2_, the value of serum iron ranged from 72.65 ± 0.50 μg/dL at 0 day to 77.42 ± 0.91 μg/dL. Values in group G_3_ and G_1_ ranged from 71.42 ± 0.60 μg/dL and 70.97 ± 0.70 μg/dL at initial level to 76.16 ± 1.09 μg/dL and 75.36 ± 1.23 μg/dL, respectively.

### Serum ferritin

Maximum value for Serum Ferritin was observed in group G_1_ at 15.24 ± 1.57 ng/mL. Following the group G_4_ (14.63 ± 2.12 ng/mL), G_2_ (14.24 ± 1.11 ng/mL), G_3_ (13.34 ± 1.30 ng/mL), and G_0_ (11.91 ± 0.12 ng/mL), as shown by Table 6.

**TABLE 6:** Effect of fortified diets on serum ferritin levels (ng/mL) among anemic women.

Treatments/Groups	Days				Means
	0	30	60	90	
G_0_	11.76 ± 0.11^e^	11.86 ± 0.08^d^	11.98 ± 0.09^e^	12.04 ± 0.17^e^	11.91 ± 0.12^c^
G_1_	13.73 ± 0.18^a^	14.09 ± 0.53^a^	16.28 ± 0.54^a^	16.88 ± 0.55^b^	15.24 ± 1.57^a^
G_2_	13.29 ± 0.31^b^	13.59 ± 0.34^b^	14.31 ± 0.37^c^	15.77 ± 0.36^c^	14.24 ± 1.11^ab^
G_3_	12.03 ± 0.09^d^	12.86 ± 0.10^c^	13.37 ± 0.14^d^	15.10 ± 0.27^d^	13.34 ± 1.30^b^
G_4_	12.67 ± 0.23^c^	13.56 ± 0.24^b^	14.77 ± 0.25^b^	17.55 ± 0.21^a^	14.63 ± 2.12^a^

Means carrying the same letters in a column are not significantly different. G_0_ = Control Group (no prebiotic given), G_1_ = 963 mg/kg Inulin + 10 ppm NaFeEDTA, G_2_ = 963 mg/kg Inulin + 20 ppm NaFeEDTA, G_3_ = 963 mg/kg GOS + 15 ppm FeSO_4_, G_4_ = 963 mg/kg GOS + 30 ppm FeSO_4_.

In terms of progression of study intervals, maximum improvement was seen in group G_4_, followed by G_1_, G_3_ and G_2_. Group G_4_ had an initial mean value of 12.67 ± 0.23 ng/mL improved to 17.55 ± 0.21 ng/mL at the end of trials. For group G_1_, the initial value of 13.73 ± 0.18 ng/mL increased to 16.88 ± 0.55 ng/mL at 90th day. Improvement in values of groups G_3_ and G_2_ were from 12.03 ± 0.09 ng/mL and 13.29 ± 0.31 ng/mL to 15.10 ± 0.27 ng/mL and 15.77 ± 0.36 ng/mL, respectively.

### Serum transferrin

**FIGURE 3:**
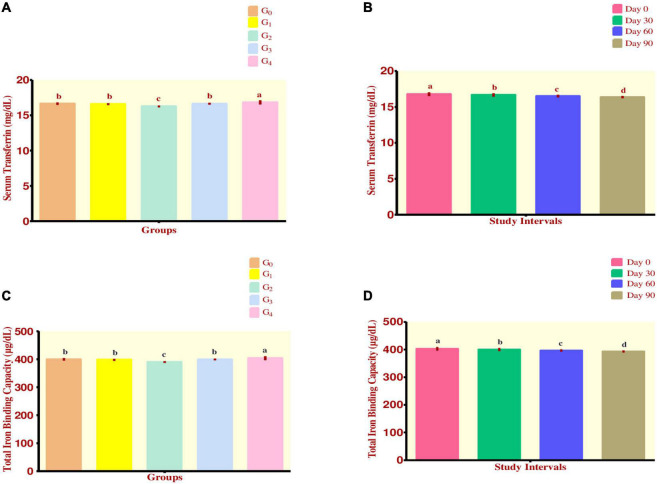
**(A–D)** Effect of fortified diets on serum transferrin and TIBC among anemic women data are presented as Means ± *SD*. *P*-value < 0.05 is considered to be significant. Means carrying the same letters are not significantly different.

From Figure 3A, maximum value for Serum Transferrin was reported in group G_4_ which was 16.82 ± 0.30 mg/dL while the minimum value was shown by group G_2_ at 16.25 ± 0.12 mg/dL. Group G_1_ had a value of 16.58 ± 0.14 mg/dL while group G_3_ recorded at 16.63 ± 0.14 mg/dL. The control group G_0_ was observed having a mean value of 16.64 ± 0.18 mg/dL.

As the study intervals progressed, it was observed that a steadily declining trend existed for the trait. Maximum decline occurred in group G_4_ which ranged from 17.15 ± 0.06 to 16.47 ± 0.12 mg/dL while minimum decline was observed in group G_2_ ranging from 16.38 ± 0.08 to 16.09 ± 0.10 mg/dL. During the efficacy trials, a significant decline in mean serum transferrin levels was observed (Figure 3B).

### Total iron binding capacity

Regarding Total Iron Binding Capacity, it was observed that maximum value was attained by group G_4_ 403.68 ± 7.27 μg/dL, followed by group G_0_ which had a value of 399.46 ± 4.35 μg/dL. Group G_3_ showed the value of 399.14 ± 3.34 μg/dL while group G_1_ had a value of 397.84 ± 3.38 μg/dL. Finally, group G_2_ was observed to be having a value of 389.80 ± 2.96 μg/dL (Figure 3C). Across the modeling trials, a significant drop in mean TIBC levels was witnessed (Figure 3D).

## Discussion

Iron as a primary component of hemoglobin plays an import role in oxygen transport and energy metabolism (21). Moreover, it is essential for several other biological functions including respiration and proliferation of cells, DNA synthesis and repair. Besides, iron is a major constituent of almost 200 enzymes involved in various metabolic functions and biochemical reactions (22, 23). Additionally, iron is involved in normal functioning of the central nervous system (CNS) and engaged in the synthesis of neurotransmitters and myelination of nerves (24, 25).

In our study, we hypothesized that cumulative effect of prebiotics and iron fortification might enhance the bioavailability of iron, thus improving body iron reserves. The results of our RCT endorsed this hypothesis and showed a significant improvement in iron absorption among iron deficiency anemic women when fed iron fortificants (either NaFeEDTA or FeSO_4_) in conjugation to prebiotics (either Inulin or Galacto oligosaccharides) as compared to the control group. Though the absorption of iron was recorded in the control group (only treated with iron fortificant), the results were more pronounced in the treatments groups. The beneficial effects of prebiotics on iron absorption can be explained through the fermentation process by gut microbiota, which helps to (i) lower the pH of the luminal content, (ii) increase in the reduction of Fe + + + to Fe + +, and (iii) facilitate the expression of mineral transport proteins in epithelial cells (26).

Regarding hemoglobin and hematocrit, it was observed that group G_3_ treated with 963 mg/kg GOS + 15 ppm FeSO_4_ had maximum hemoglobin and hematocrit levels at the end of 90 days trial. It has been reported that bioavailability of ferric form is 3–4 times less compared to that of ferrous form due to poor solubility of ferric form in alkaline media (27). Therefore, maximum increase in Hb and hematocrit levels were seen in groups treated with FeSO_4_ which contains iron in ferrous form as compared to NaFeEDTA that contains iron in ferric form.

In agreement to the previous studies of Ahmad et al. and Gershoff et al. (10, 28), our study observed that serum iron levels were significantly improved in all the groups of anemic women when fed with iron fortificants and prebiotics, compared to the control group. Furthermore, maximum values of both RBC count and MCV levels were reported in group G_4_ fed with 963 mg/kg GOS and 30 ppm FeSO_4_ as compared to other treatment groups.

Hb levels are usually recommended for detection of iron deficiency anemia, despite that serum ferritin is a more reliable indicator used to detect iron deficiency anemia (29). Ferritin is considered to be the storage form of iron in the body and falls early in case of iron deficiency anemia (30). In our study, serum ferritin levels were considerably improved among anemic women at the end of the trial period, in accordance to a previous study conducted by Thuy et al. (31). Besides, studies have suggested that serum transferrin can be a useful indicator for diagnosis of iron deficiency anemia, highly sensitive and specific in nature (32).

In our trials, a significant decrease in serum transferrin levels was observed over a period of 90 days among all the treatment groups, with maximum decline occurring in group G_2_ (963 mg/kg Inulin + 20 ppm NaFeEDTA). The reason behind can be explained that serum transferrin has been known to increase in anemic individuals, indicating that more iron is needed by the body. On the other hand, as soon as anemia starts to improve, serum transferrin levels of an individual are declined (33). Similarly, TIBC is defined as the total amount of iron in the body which can be carried by transferrin. Transferrin is a protein found in the body which is responsible to carry iron throughout the body (30). TIBC levels are supposed to decline when anemia is being improved in patients as shown in our results. Lobo et al. on the similar lines conducted a study in 2011 and concluded that there was a steady decrease in TIBC levels over the course of trials, which indicated that iron reserves were being improved among anemic subjects (34).

## Conclusion

In conclusion, our study revealed that cumulative diet containing prebiotics and iron fortificants improved levels of hemoglobin, hematocrit, RBC count, MCV, serum iron, serum ferritin, serum transferrin, and TIBC. These effects were more pronounced among treatment groups as compared to the control group (receiving only iron fortificants) highlighting that the addition of prebiotics appeared to increase absorption of iron among anemic women.

## Data availability statement

The raw data supporting the conclusions of this article will be made available by the authors, without undue reservation.

## Ethics statement

This study was conducted according to the guidelines from the Declaration of Helsinki. All procedures involving human subjects/patients were approved by the Institutional Review Committee (IRC) for Biomedical Research of the University of Veterinary and Animal Sciences, Lahore (Reference No. 037/IRC/BMR). Moreover, informed written consent was taken from all the study subjects prior to recruiting them to the RCT. The trial was also registered at clinicaltrials.gov with ID number “NCT03894449” (https://clinicaltrials.gov/ct2/show/NCT03894449).

## Author contributions

AA, WA, RA, and UF conceptualized the study. SZ, RA, and AA performed the data collection and analysis. WA, JA, HS, and AA wrote the initial manuscript. SA, MG, and JA proofread the manuscript. SI did the final editing. All authors contributed to the article and approved the submitted version.
