# Supplementary figures and images for: Effect of inulin, galacto oligosaccharides and iron fortification on iron deficiency anemia among women of reproductive age; a randomized controlled trial
Source: Front Nutr. 2022 Nov 14;9:1028956. doi: 10.3389/fnut.2022.1028956 (PMC9702533; doi:10.3389/fnut.2022.1028956)

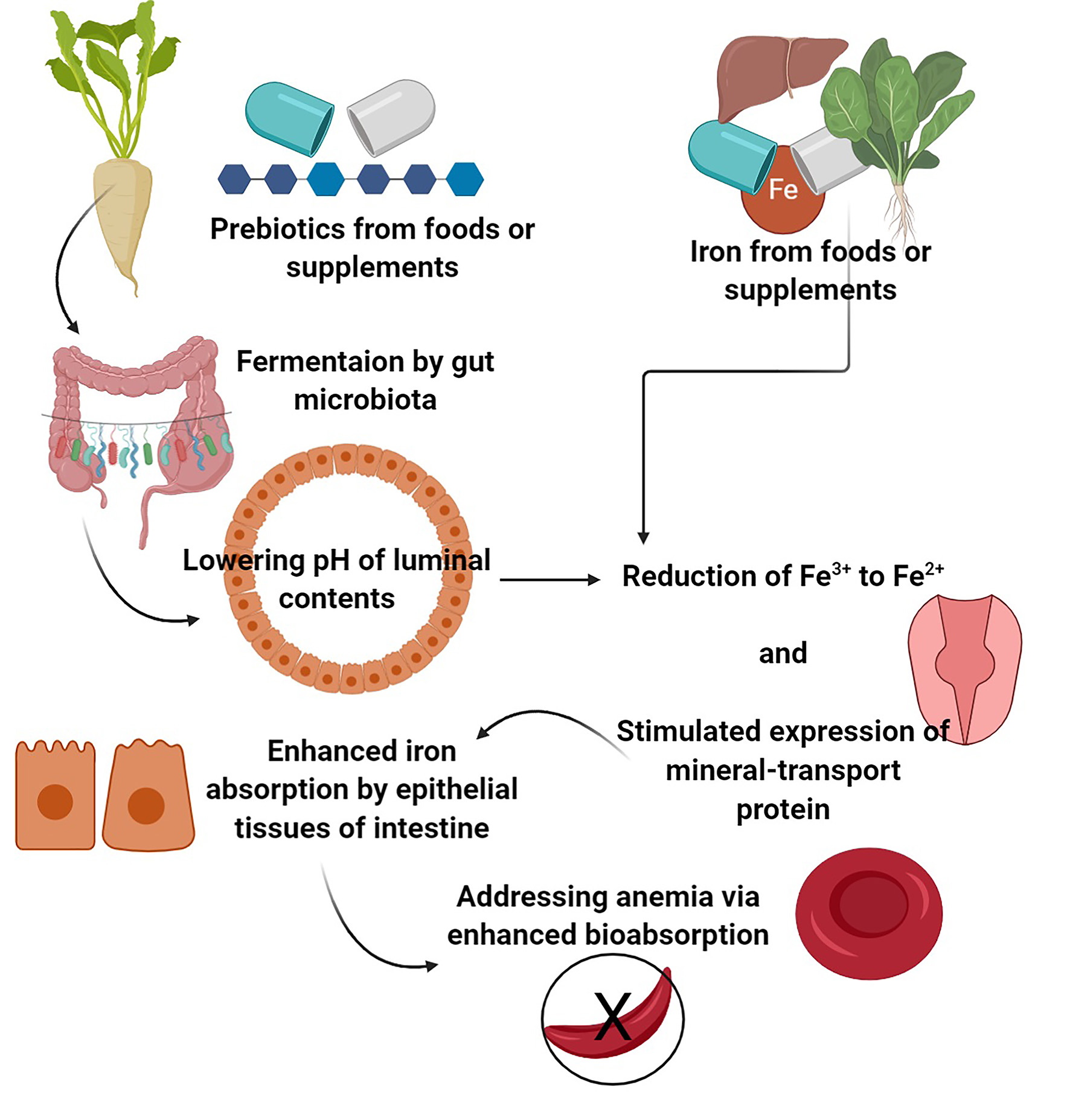

Supplement: Supplementary file 1 [file Image_1.JPEG]
